# Effects of in ovo delivered xylo- and mannan- oligosaccharides on growth performance, intestinal immunity, cecal short-chain fatty acids, and cecal microbiota of broilers

**DOI:** 10.1186/s40104-021-00666-z

**Published:** 2022-02-08

**Authors:** Amit Kumar Singh, Utsav Prakash Tiwari, Birendra Mishra, Rajesh Jha

**Affiliations:** Department of Human Nutrition, Food and Animal Sciences, University of Hawaii at Manoa, 1955 East-West Rd, Honolulu, HI 96822 USA

**Keywords:** Broilers, Gut health, Immunity, in ovo, Mannanoligosaccharides, Microbiota, Oligosaccharides, Prebiotics, Xylooligosaccharides

## Abstract

**Background:**

This study investigated a novel in ovo feeding strategy to determine the prebiotic effects of xylo- and mannan- oligosaccharides (XOS and MOS) differing in the degree of polymerization. A total of 192 fertilized eggs were divided into 6 treatment groups: i) normal saline control (NSC), ii) xylotriose (XOS3), iii) xylotetraose (XOS4), iv) mannotriose (MOS3), v) mannotetraose (MOS4), and vi) no injection control (NIC), each containing 4 replicate trays with 8 eggs per replicate. On d 17 of incubation, 3 mg of oligosaccharides (except for controls) dissolved in 0.5 mL of 0.85% normal saline were injected into the amnion of Cobb 500 broilers eggs. After hatch, the chicks were raised for 28 d under standard husbandry practices and were fed a commercial broilers diet ad libitum*,* and samples were collected periodically.

**Results:**

The hatchability, growth performance, and relative weights of breast, drumstick, liver, and proventriculus were not different among the treatments (*P* > 0.05). The XOS3 injection increased the total short-chain fatty acid production at d 28 compared with both control groups (*P* < 0.05). The villus height to crypt depth ratio was significantly higher in the XOS4 group than both controls on the hatch day (*P* < 0.01) but were not different among any treatments on d 7 and 28 (*P* > 0.05). On the hatch day, the expression level of the *CD3* gene (a T cell marker) was increased by XOS3, while the *IL-10* gene (a marker of anti-inflammatory cytokine) was reduced by MOS4 (*P* < 0.05) compared with both controls. Compared with both controls, XOS3 exhibited a trend of reduction for *IL-10* (*P* = 0.074). No cytokines or lymphocyte markers were affected by the treatments on d 7 (*P* > 0.05), except XOS4 increased *IL-4* compared with NSC (*P* < 0.05). The broilers in the MOS4 group had higher operational taxonomic units (OTUs) and had more differentially abundant taxa, including order Lactobacillales and family Leuconostocaceae (*P* < 0.05) than both controls on d 28. The predictive functional profiling indicated that the linoleic acid metabolism pathway was enriched in the cecal microbiota of the XOS3 group compared with both controls (*P* < 0.05).

**Conclusion:**

The effects of these XOS and MOS on ileal mucosa and immunity are transient, but the effects on fermentation and cecal microbiota are prolonged, and further research is warranted to determine their use as a gut health promoter in poultry.

**Supplementary Information:**

The online version contains supplementary material available at 10.1186/s40104-021-00666-z.

## Introduction

Poultry productivity depends on the combined effects of several factors such as the level of nutrients in feed, proper management practices, and the health status of the birds. To keep the enteric infections under control and promote growth, the supplementation of antibiotics in the poultry feed has been a regular practice. However, the use of antibiotic growth promoters (AGP) is restricted or banned in several countries due to the public health concern of antibiotic resistance. Consequently, there is a growing demand for alternatives to AGPs, and some products have shown potential in improving production while others require additional research. Several alternatives like organic acids, phytogenic compounds, direct-fed microbials, probiotics, enzymes, and prebiotics are applied in feed or water to generate similar benefits as AGPs [1, 2]. Several oligosaccharides that are not digested by the host’s endogenous enzymes but are rapidly fermented by the microbiota in the hindgut are supplemented in the feed of broilers to achieve the prebiotic benefits [2, 3].

The xylo-oligosaccharides (XOS) added to the broiler feed have been described to improve the growth by stimulating butyrate-producing bacteria through cross-feeding of lactate [4]. The feeding of XOS to laying birds has been found to increase *Bifidobacterium* and short-chain fatty acids (SCFA) in ceca and enhance immunoglobulin A (IgA), IgM, and tumor necrotic factor-alpha (*TNF-ɑ*) [5]. Likewise, the feeding of mannan-oligosaccharides (MOS) in the regular broilers diet has increased *Lactobacillus* community diversity and decreased *Clostridium perfringens* and *E. coli* in the ileum [6]. The yeast cell wall extracted MOS has been used frequently in poultry feeding because of its known benefit of reducing the pathogens’ attachment to the host intestinal epithelial cells by binding to the mannose-binding lectins of Gram-negative bacteria expressing type-1 fimbriae [7, 8]. These oligosaccharides could be utilized as substrates by commensal bacteria to yield SCFA. The SCFA is known to interact with the antigen-presenting cells such as dendritic cells, macrophages, and T cells, along with gut-associated epithelial cells to regulate the immune cells [9].

Prebiotics are also injected in ovo and have been reported to increase the villus height to crypt depth ratio and enhance innate and adaptive immunity in broilers [10–12]. In ovo feeding is a precision nutritional strategy to support the hatching chicks’ adaptation during the transition from the yolk-based resources to other nutrients [13]. In ovo administration of wheat-based prebiotics on d 17 has been found to increase the intestinal population of beneficial bacteria such as *Lactobacilli* and *Bifidobacteria* [14]. During 17/18th day of incubation, the chicken embryo is fully developed and can utilize the nutrients supplied via amnion. The late-term chicken embryo can swallow the prebiotics injected into their amnion, and it can easily pass to their intestinal tract for interaction with the intestinal immune cells and proliferating gut microbiota.

Dietary components are in intimate contact with the immune system in the intestine, and the presence of nutrients in the intestine may be necessary for the proper development and function of gut-associated lymphoid tissue [15]. The residual feed components in the digesta of the birds are also required to provide the substrate and the source of energy to the gut microbiota. The gastrointestinal tract (GIT) of chicken gets rapidly colonized by microbes after hatch and could reach the maximum bacterial population density within the first three days post-hatch [16, 17]. This rapid establishment of gut microbiota essentially indicates the early window of opportunity to nutritionally modulate its colonization as the pH and anaerobic environment would become dominant with the increasing age of the broilers [2, 18].

So far, the effects of feeding variable subunits of XOS and MOS to the broiler’s embryo via in ovo injection have not been reported in the literature. Therefore, the objectives of this experiment were to evaluate the effects of in ovo injection of XOS and MOS with differing degrees of polymerization on the growth performance, immune modulation, cecal SCFA production, and cecal microbiota diversity in broilers.

## Materials and methods

All animal care procedures were approved by the Institutional Animal Care and Use Committee of the University of Hawaii.

### in ovo feeding

A total of 192 fertile eggs (Cobb 500) on the 17th day of incubation from the 35-week breeding flock was obtained from a commercial hatchery (Asagi Hatchery Inc., Honolulu, HI, USA). The eggs were incubated at 37.5 °C and relative humidity of 58% in an incubator (GQF incubator, Savannah, GA, USA). After the eggs were acclimatized in the incubator for > 8 h, they were randomly assigned to 24 sections (4 replicates with 8 eggs) of egg holder flat trays. Next, 4 replicate sections were randomly assigned to each of six treatments (*n* = 32 eggs/treatment). After acclimatization, the eggs from each replicate section were taken out in a biosafety cabinet on d 17 for in ovo injection and were placed back to the incubator within 15 min. The broad end site of all eggs was disinfected with 10% povidone-iodine solution, and a tiny punch hole (shell perforation) was made using a stabbing awl with a fixed 1 mm depth. After every punch, the tip of the awl was disinfected with 70% ethanol and wiped with sterile gauze. The solution was injected in the amniotic sac of each egg using a blunt tip 21-gauge sterile needle inserted to 2.80 cm length from the longest axis through the broad end and passing beyond the air sac.

All the eggs were sealed using non-toxic glue. Each oligosaccharide (Megazyme International Ireland Ltd. Bray, Ireland) treatment (xylotriose (XOS3), xylotetraose (XOS4), mannotriose (MOS3), and mannotetraose (MOS4)) was prepared at a concentration of 6 mg/mL in 0.85% normal saline and was injected 0.5 mL per egg. In total, there were six treatment groups: 1) 0.85% normal saline control (NSC), 2) 0.5 mL 0.85% normal saline containing 3 mg XOS3, 3) 0.5 mL 0.85% normal saline containing 3 mg XOS4, 4) 0.5 mL 0.85% normal saline containing 3 mg MOS3, 5) 0.5 mL 0.85% normal saline containing 3 mg MOS4, and 6) no injection control (NIC). The incubated eggs were later transferred to a hatcher (GQF incubator, Savannah, GA, USA) set at 37 °C and relative humidity of 75% on d 19 following the instructions for pre-set hatcher. Each replicate group of eggs from the setter was again randomly assigned to one of 24 compartments in 6 hatcher trays separated by the dividers.

### Post-hatch chicks management, growth performance, and organs relative weight

After the hatching of all pipped eggs, the unhatched eggs were counted and opened to check the cause of the embryonic death to rule out any infection or injury-related deaths, and then hatchability was calculated for each treatment. At day 21of incubation, most of the eggs in all the treatments group hatched within 12 h, and they were included in further growth performance study. The eggs that hatched late were included in the hatchability calculation, but the chicks were excluded from further experimentation. The chicks were weighed, tagged, and placed randomly in 30-floor pens (5 birds per pen), making 5 replicates of each treatment. All birds were kept on floor pens covered with wood shavings and were raised in a standard environment (light, temperature, and humidity) recommended for commercial broilers. The temperature was maintained at 35 °C in the first week and gradually reduced to 28 °C by the third week. During the entire 28 d post-hatch trial period, the birds were fed a commercial corn-soybean meal-based pellet diet (Table 1). The birds had unrestricted access to water and were fed ad libitum. The feed consumption and the bodyweight of the birds were measured in each pen at 7, 14, 21, and 28 days of age. The average daily gain (ADG), average daily feed intake (ADFI), and feed conversion ratio (FCR) were calculated from weekly feed intake and body weight data. The feed wastage and bird mortality were recorded daily, and the feed consumption and FCR were adjusted for feed wastage and remaining birds. On d 28, five birds per treatment (1 bird per pen) were selected randomly to determine organ weights after euthanizing with CO_2_ gas. The weight of breast muscle, drumsticks, gizzard, and proventriculus were recorded, and the relative weight (% of live body weight) was calculated.

**Table 1:** Nutrient composition of commercial starter diet fed to broilers from d 0–28 post-hatch (as-fed basis, g/kg unless indicated)

Item	Inclusion level
Crude Protein (Min)	220.0
Lysine (Min)	10.0
Methionine	4.5
Crude Fat (Min)	35.0
Crude Fiber (Min)	40.0
Calcium (Ca) (Min)	9.0
Calcium (Ca) (Max)	14.0
Phosphorus (P) (Min)	6.0
Salt (NaCl) (Min)	3.0
Salt (NaCl) (Max)	8.0
Total Selenium (Se) (Min)	0.60 ppm
Total Selenium (Se) (Max)	0.72 ppm
Phytase (*A. oryzae*) (Min)	500 FYT/kg
Analyzed gross energy, MJ/kg	15.90

One phytase unit (FYT) liberates one micromole of inorganic phosphorus per minute from sodium phytate at pH 5.5, and 98.6 F. Contains a source of phytase, Ronozyme HiPhos GT, which can hydrolyze phytate, increasing the digestibility of phosphorus in diets containing phytate-bound phosphorus

### Ileal and cecal sample collection

A section of the mid-ileum (approximately 2–3 cm) was excised and flushed with 1 × phosphate-buffered saline (PBS) and then fixed in 10% neutral buffered formalin (NBF). The ileum was identified as the segment 1 cm distal to Meckel’s diverticulum and 1 cm anterior to the ileocecal junction. The samples for ileal histology were collected from 4 birds per treatment (1 bird per compartment) on d 0 and 5 birds per treatment (1 bird per pen) on d 7, 21, and 28. A small section (50–100 mg) of the flushed ileum was cut and collected in cryovials and snap-frozen in liquid nitrogen on d 0 and 7 and was stored at − 80 °C until RNA extraction. On d 28, cecal contents from each pen were collected in a 15-mL sterile centrifuge tube on ice. The cecal digesta was mixed with the cut pipette tip, and a small amount (400–500 mg) was transferred to a cryovial for snap freezing and storing at − 80 °C for bacterial DNA extraction. The 15-mL centrifuge tube with the remaining cecal contents was snap-frozen and stored at − 20 °C for later analysis of SCFA.

### Histomorphometry of ileal mucosa

The ileal tissues fixed in 10% NBF were passed through a series of ethanol, embedded in paraffin wax, sectioned into 5 μm thickness, and stained with Hematoxylin and Eosin (H&E) as previously described [1]. The stained slides were observed under 8 × objective of an upright microscope (Olympus BX43, Olympus Co, Tokyo, Japan), and the villus height (VH) and crypt depth (CD) were recorded using Infinity Analyze software (Lumenera Corporation, Ottawa, ON, Canada) image processing and analysis system. The VH was measured from the tip of mucosal projection to the valley between two VH, and the CD was measured from the invagination opening to its base above the lamina propria lying above lamina muscularis. The VH and CD were recorded from three sets of each section (18 sets per replicate). The average measurement was taken from all 18 readings per replicate for VH, CD, and their ratio (VH:CD) was calculated. The histomorphometry data are presented as average for each treatment along with their standard error.

### Gas chromatographic determination of short-chain fatty acids

Approximately 1 g of cecal digesta was mixed with 1 mL of distilled water (1:1, w/v) by vortexing in a centrifuge tube and centrifuged at 10,000 × *g* for 5 min at 4 °C to precipitate the solid contents. Following centrifugation, 400 μL of supernatant was transferred into a new microcentrifuge tube containing 100 μL of 25% metaphosphoric acid and 100 μL of 48.95 mmol/L trimethylacetic acid (TMA), and the final volume was made 1500 μL by adding distilled water. The major SCFA was analyzed by the gas chromatography (Trace 1300, Thermo Scientific, Waltham, MA, USA) equipped with AS 1310 series automatic liquid sampler and a flame ionization detector as previously described by Singh et al. [19]. A calibration curve of the external standard was prepared in the range of 0 to 8 mmol/L from supplied SCFA mix (Sigma-Aldrich, St. Louis, MO, USA). For both samples and external standards, TMA (Sigma-Aldrich, St. Louis, MO, USA) was included as an internal standard. The calibration curve was generated based on the response ratio of external to internal standards. The data handling and chromatogram processing was done on Chromeleon™ 7.2 software (Thermo Scientific, Waltham, MA, USA).

### Quantitative real-time PCR (qPCR) assay for gene expression

Total RNAs from ileal tissues collected on d 0 and d 7 were isolated using TRIzol® reagent (Invitrogen, Carlsbad, CA, USA) according to the manufacturer’s instruction. Reverse transcription was performed using the high-capacity cDNA Reverse Transcription kit as previously described [20]. The primers for immune genes (innate and adaptive immune systems) were designed as used previously [10]. The qPCR amplification conditions were set as 50 °C for 2 min, 95 °C for 2 min followed by 40 cycles of 95 °C for 15 s for denaturation, 60 °C for 15 s for annealing, and 72 °C for 1 min for the extension. After 40 cycles, melt curves were produced to check the specificity of the used primers. Beta-actin (β-actin) was used as an endogenous reference gene and analyzed in triplicate. The target genes (*CD3, CD56, chB6, IL-4, IL-10 IL-12, IL-1β,* and *TLR-4*) were analyzed in duplicate, and the average of all observations was taken for both the reference and the target genes replicate samples. The cycle of threshold (Ct) generated for each gene transcript after the qPCR run was recorded to compare the gene expression of the oligosaccharide treatments with NIC. The relative abundance of the target genes was normalized to the reference gene. The relative gene expression of the target gene was calculated as base two exponential delta-delta Ct (2^ΔΔCt^) as previously described [21, 22]. The mean ΔCt of NIC was used as a control to calculate the ΔΔCt value for each treatment replicate.

### 16S rRNA amplicon sequencing and bioinformatics

The microbial DNA from the cecal digesta of broilers was extracted using QIAamp® Fast DNA Stool Mini Kit (Qiagen, Hilden, Germany) according to the manufacturer’s instructions. The 16S rRNA gene amplicon library was prepared by targeting V3–V4 variable regions of the 16S rRNA gene in the PCR amplification. The primer used for amplification contained an Illumina overhang adapter attached to the locus-specific primer, and the forward sequence was.

5′-TCGTCGGCAGCGTCAGATGTGTATAAGAGACAGCCTACGGGNGGCWGCAG-3′ and reverse primer sequence was.

5′-TCTCGTGGGCTCGGAGATGTGTATAAGAGACAGGACTACHVGGGTATCTAATCC-3′ [23]. The amplicon PCR, purification, and the addition of Nextera XT dual indices to the amplicon were performed as previously described [19]. The libraries were quantified using the Quant-iT PicoGreen dsDNA Assay Kit, normalized, and pooled. The normalized and pooled amplicons were sequenced on the Illumina MiSeq desktop sequencer (2 × 300 bp paired-end run) at the University of Hawaii at Manoa Advanced Studies in Genomics, Proteomics, and Bioinformatics core facility.

For processing the paired-end forward and reverse reads, the demultiplexed sequences were imported in the Quantitative Insights Into Microbial Ecology (QIIME™ version 2.0 release 2021.2), and Qiime2 pipelines and plugins were utilized for the downstream analysis [24]. Once the paired-end sequence reads were imported, the Divisive Amplicon Denoising Algorithm2 (DADA2) pipeline was applied to denoise, trim, and filter these sequences. After denoising and filtering, the unrooted and rooted tree was generated for phylogeny using the align-to-tree-mafft-fasttree pipeline. For the taxonomical analysis, a Naïve Bayes classifier pre-trained on the Greengenes 13_8 99% operational taxonomic unit (OTU) was included. The alpha and beta diversity were analyzed by the diversity plugin using the core-metrics-phylogenetic method with a sampling depth of 20,000 frequencies per sample. The alpha diversity providing information about the richness was visualized via observed OTUs, and the evenness of OTUs was represented by Shannon Index. The observed OTUs were further visualized using the Venn diagram to show the shared and unique OTUs present in each treatment group. The taxonomic table collapsed at species level was further analyzed on Galaxy for linear discriminant analysis (LDA) using linear discriminant analysis effect size (LEfSe) tools. We also accessed the Clusters of Orthologous Groups of proteins (COGs) and the Kyoto Encyclopedia of Genes and Genomes (KEGG; Uji, Kyoto, Japan) databases and used Phylogenetic Investigation of Communities by Reconstruction of Unobserved States (PICRUSt) to determine the effect of in ovo oligosaccharides on the predictive functional profile of cecal microbiota [25, 26]. A closed reference OTU from the Greengenes database was supplied for comparison of the sample features. The obtained data was analyzed for the relevant functional profile in the oligosaccharides treatments compared with NSC and NIC groups using a software package Statistical Analysis of Taxonomic and Functional Profiles version 2.1.3 to present the mean difference and the confidence intervals [27].

### Statistical analysis

All the variables were compared among treatments using the MIXED procedure of SAS v9.4 (SAS Institute Inc., Cary, NC, USA) for hatchability, growth performance, organ weight, histomorphometry, and SCFA data. Hatchability was noted for all replicates, and it was subjected to statistical analysis after arcsine square root transformation. Differences among treatment means were considered significant at *P* < 0.05. Significant differences between treatment means were separated by the Tukey test using the pdmix macro of SAS. For immune gene expression data, all the relative abundance was log-transformed, and test variables were compared with control variables by the t-test procedure of SAS. For analysis of microbiota diversity, Kruskal-Wallis pairwise test for alpha diversity and pairwise PERMANOVA for beta diversity were performed in QIIME2. Wilcoxon rank-sum test was used for analyzing log relative abundance of differential species and was considered significant at *P* < 0.05. The bar diagram and the box and whisker plot were generated using ggplot2, the principal coordinate biplot was created by phyloseq, the Venn diagram was plotted using ggvenn, and the abundance heatmap was generated by pheatmap package in R v4.1.0. For presenting histogram and cladogram, statistical analyses were performed using linear discriminant analysis effect size (LEfSe) on Galaxy web application at a significance of *P* < 0.05. White’s non-parametric t-test was run to analyze the predicted functional pathways [28]. The Spearman’s correlation and the principal component analysis were generated using JMP pro v14.1.0 (SAS Institute Inc., Cary, NC, USA).

## Results

### Growth performance and organs relative weight

The hatchability was not different (*P* > 0.05) across treatments (Table 2). The growth performance parameters (ADFI, ADG, and FCR) during 0–28 post-hatch were not different (*P* > 0.05) among treatments (Table 3). The in ovo injection of oligosaccharides did not affect the relative weight of liver, proventriculus, drumstick, and breast compared to both controls (*P* > 0.05) (Table 4). However, the relative weight of the gizzard was different across treatments, and it was lowest in the XOS3 group and highest in the MOS4 group (*P* < 0.05).

**Table 2:** Hatchability of eggs on d 21 of incubation in response to in ovo treatments

Treatments	%, Hatch
Normal saline	79.3
Xylotriose	93.6
Xylotetraose	85.6
Mannotriose	82.9
Mannotetraose	82.0
No-injection	89.7
SEM (*n* = 4)	8.67
*P*-value	0.86

**Table 3:** Effects of in ovo injection of oligosaccharides on the growth performance of broilers from d 0 to 28 post-hatch

Variables	Treatments							
	NSC	XOS3	XOS4	MOS3	MOS4	NIC	SEM (*n* = 5)	*P*-value
ADFI, g/d								
0–7 d	18	19	19	18	18	19	0.58	0.215
0–21 d	59	59	57	55	57	60	1.25	0.191
0–28 d	80	80	76	77	78	80	1.68	0.427
ADG, g/d								
0–7 d	15	16	16	15	15	16	0.40	0.300
0–21 d	43	44	42	42	43	43	1.07	0.604
0–28 d	54	55	51	55	54	54	1.56	0.690
FCR								
0–7 d	1.20	1.20	1.17	1.17	1.18	1.18	0.04	0.996
0–21 d	1.37	1.34	1.37	1.32	1.32	1.40	0.03	0.137
0–28 d	1.49	1.48	1.48	1.41	1.45	1.48	0.04	0.747
FBW, g/bird	1550	1569	1477	1572	1555	1556	43.26	0.666

*ADFI* average daily feed intake, *ADG* average daily gain, *FCR* feed conversion ratio, *FBW* final body weight, *NSC* normal saline, *XOS3* xylotriose, *XOS4* xylotetraose, *MOS3* mannotriose, *MOS4* mannotetraose, *NIC* no injection control

**Table 4:** Effects of in ovo injection of oligosaccharides on relative organ weight (g/100 g of carcass weight) of digestive organ and meat portions at d 28 post-hatch

Variables	Treatments							
	NSC	XOS3	XOS4	MOS3	MOS4	NIC	SEM (*n* = 5)	*P*-value
Liver	3.12	2.95	3.06	3.04	3.15	2.89	0.11	0.557
Proventriculus	0.63	0.59	0.64	0.68	0.61	0.60	0.04	0.737
Gizzard	1.99^ab^	1.90^b^	2.21^ab^	2.23^ab^	2.28^a^	2.20^ab^	0.08	0.015
Drumstick	4.30	4.50	4.47	4.44	4.08	4.18	0.20	0.580
Breast	21.72	21.29	20.46	20.52	22.07	22.07	0.78	0.516

*NSC* normal saline, *XOS3* xylotriose, *XOS4* xylotetraose, *MOS3* mannotriose, *MOS4* mannotetraose, *NIC* no injection control

^a-b^Within rows for variables, means without a common superscript differ (*P* < 0.05)

### Gut health parameters

The VH and CD of ileum were not different (*P* > 0.05) across treatments on the hatch day and d 7, 21, and 28 post-hatch (Table 5). The VH:CD ratio was not different across treatments on d 7, 21, and 28 post-hatch (*P* > 0.05). However, the VH:CD ratio was significantly different (*P* < 0.01) on the hatch day. The VH:CD ratio was highest for the XOS4 group and lowest for NIC. The production of cecal acetate, butyrate, and total SCFA on d 28 post-hatch was different (*P* < 0.05) across treatments (Table 6). The in ovo injection of XOS3 enhanced the production of cecal acetate, butyrate, and total SCFA compared to both controls.

**Table 5:** Effects of in ovo injection of oligosaccharides on ileum morphology of broiler chickens at hatch day, and d 7, 21, and 28 post-hatch

	Treatments							
Parameters	NSC	XOS3	XOS4	MOS3	MOS4	NIC	SEM (*n* = 5)	*P*-value
Hatch day								
VH, μm	371.0	410.8	405.6	412.9	421.8	330.7	27.5	0.232
CD, μm	69.5	58.7	53.4	54.9	71.0	65.8	5.1	0.128
VH:CD	5.5^bc^	7.1^abc^	7.8^a^	7.6^ab^	6.9^abc^	5.1^c^	0.5	0.006
7 d post-hatch								
VH, μm	533.7	475.8	517.0	492.2	486.0	502.3	29.2	0.752
CD, μm	81.4	78.5	84.5	78.8	79.4	85.0	2.7	0.362
VH:CD	6.6	6.1	6.2	6.3	6.2	6.0	0.3	0.854
28 d post-hatch								
VH, μm	793.2	936.0	861.0	919.4	804.8	875.7	52.3	0.323
CD, μm	99.4	104.8	99.7	103.8	103.0	101.5	2.7	0.659
VH:CD	8.1	9.0	8.8	9.0	8.0	8.7	0.5	0.475

*VH* villus height, *CD* crypt depth, *VHCD* villus height to crypt depth ratio

*NSC* normal saline, *XOS3* xylotriose, *XOS4* xylotetraose, *MOS3* mannotriose, *MOS4* mannotetraose, *NIC* no injection control

^a-c^Within rows for parameters, means without a common superscript differ *(P* < 0.05)

**Table 6:** Effects of in ovo injection of oligosaccharides on cecal short-chain fatty acids (mmol/kg wet digesta) in broilers at d 28 post-hatch

	Treatments							
Variables	NSC	XOS3	XOS4	MOS3	MOS4	NIC	SEM (*n* = 5)	*P*-value
Acetate	51.4^b^	68.1^a^	61.5^ab^	58.9^ab^	57.8^ab^	51.0^b^	3.50	0.019
Propionate	5.9	6.4	6.2	5.9	6.1	6.9	1.34	0.995
Butyrate	6.2^b^	11.1^a^	8.8^ab^	6.1^b^	7.0^b^	7.1^b^	0.92	0.006
Total SCFA	66.1^b^	88.3^a^	79.5^ab^	73.3^ab^	73.5^ab^	67.8^b^	4.31	0.015

*NSC* normal saline, *XOS3* xylotriose, *XOS4* xylotetraose, *MOS3* mannotriose, *MOS4* mannotetraose, *NIC* no injection control

^a-b^Within rows for variables, means without a common superscript differ *(P* < 0.05)

**Fig. 1:**
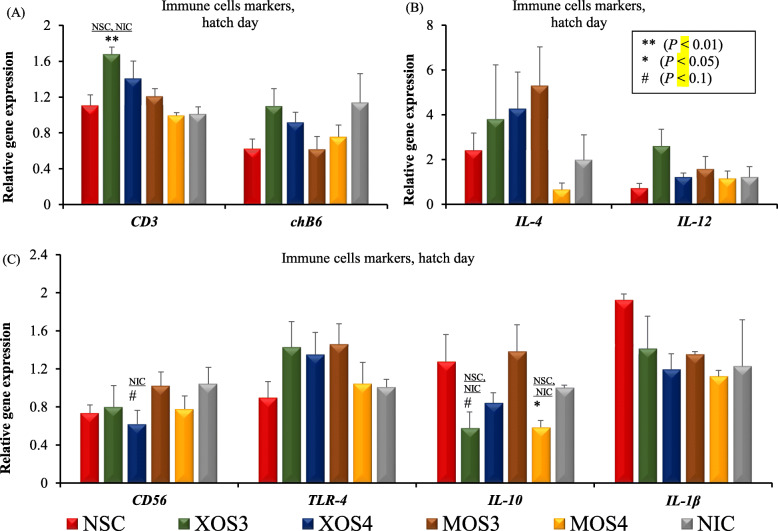
Effects of in ovo injection of oligosaccharides on gene markers of immune cells of broilers on the hatch day. The expression of each gene was examined using RT-qPCR and expressed as the normalized abundance relative to β-actin reference gene compared with no injection control group. NSC: normal saline, XOS3: xylotriose, XOS4: xylotetraose, MOS3: mannotriose, MOS4: mannotetraose, NIC: no injection control

**Fig. 2:**
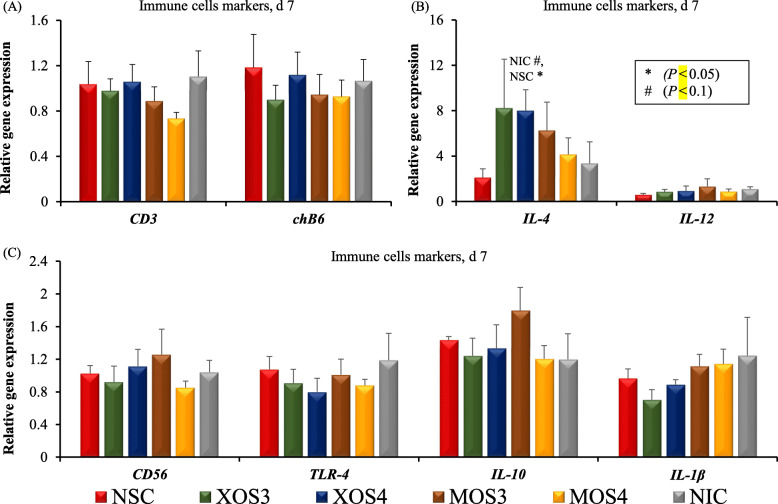
Effects of in ovo injection of oligosaccharides on gene markers of immune cells of broilers on d 7 post-hatch. The expression of each gene was examined using RT-qPCR and expressed as the normalized abundance relative to β-actin reference gene compared with no injection control group. NSC: normal saline, XOS3: xylotriose, XOS4: xylotetraose, MOS3: mannotriose, MOS4: mannotetraose, NIC: no injection control

**Fig. 3:**
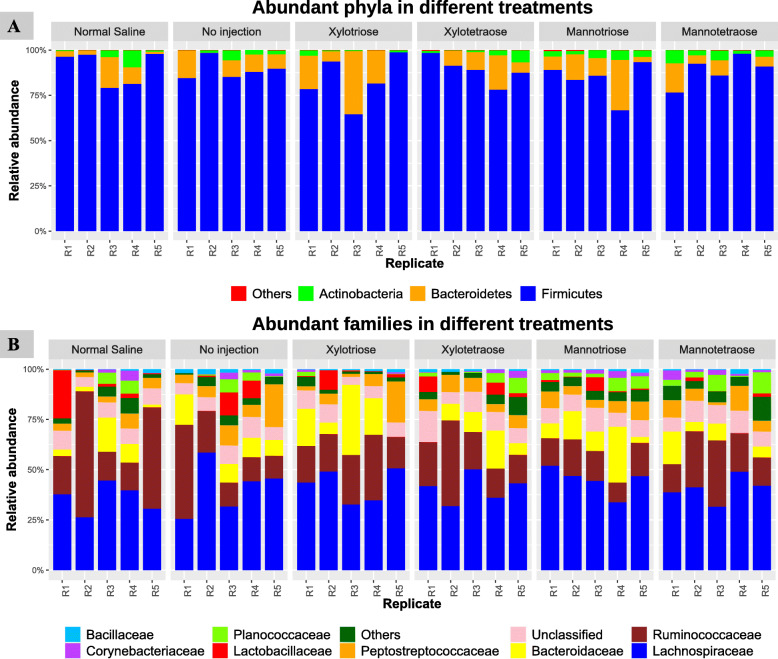
Taxonomic composition of the cecal microbiota of broilers on d 28 at **A)** phylum level, and **B)** at family level in different treatments. ‘Others’ refer to the minor bacterial phyla whose average relative abundance were < 1%. R1–R5 represents replicate samples from each experimental unit. The control is no injection treatment group. “Unclassified” refers to those bacterial taxa that were only assigned at a higher taxonomic level. The column labels R1–R5 denote replicate samples in each treatment

**Fig. 4:**
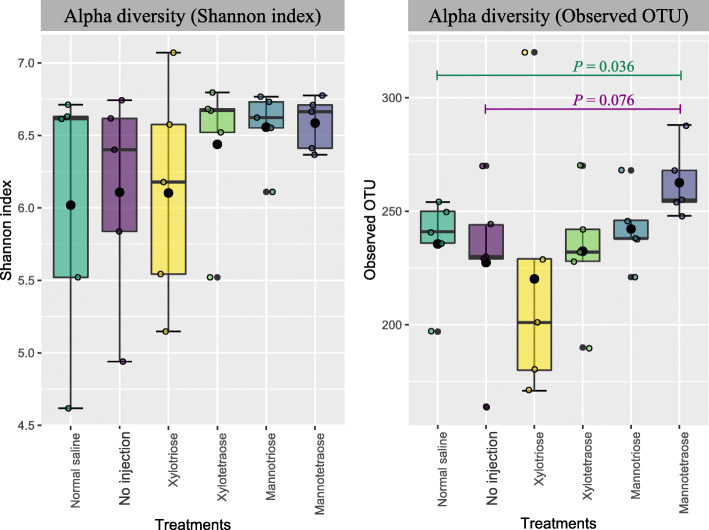
Box and whisker plots showing alpha diversity analysis based on Shannon index and observed OTUs of different treatments at 20,000 reads depth per sample of cecal contents of broilers at day 28 post-hatch in response to in ovo injection. The middle line of the box and whisker plot represents the median, the lower and upper hinges denote 1st and 3rd quartiles, respectively. The whiskers extending from the box show the highest and lowest value that falls within 1.5 times of the interquartile range. The black dots are the mean, while the colored circles represent individual values. The statistical significance was determined by the non-parametric Kruskal-Wallis test. The control is the no injection treatment group

On hatch day, the expression level of the *CD3* gene (a T cell marker) was increased by XOS3, while the level of the *IL-10* gene (a marker of anti-inflammatory cytokine) was decreased by MOS4 (*P* < 0.05) compared with both controls (Fig. 1). The birds in the XOS3 group had a trend (*P* = 0.074) on the reduction of *IL-10* compared with both controls. The immune markers of T cells, B cells, proinflammatory cytokines, and anti-inflammatory cytokines were not expressed differently (*P* > 0.05) across treatments on d 7 post-hatch (Fig. 2). The d 28 cecal microbiota analysis revealed that the Firmicutes was the most abundant phyla, followed by the Bacteroidetes across all the treatments (Fig. 3a). Likewise, Lachnospiraceae was the most abundant family across all the treatment groups, while Ruminococcaceae was the second most abundant (Fig. 3b). In ovo injection of MOS4 increased the frequency of observed OTUs compared with NSC (*P* < 0.05) and exhibited a trend (*P* = 0.076) of increased OTUs compared with NIC for cecal microbiota analyzed from cecal contents of d 28 post-hatch (Fig. 4).

**Fig. 5:**
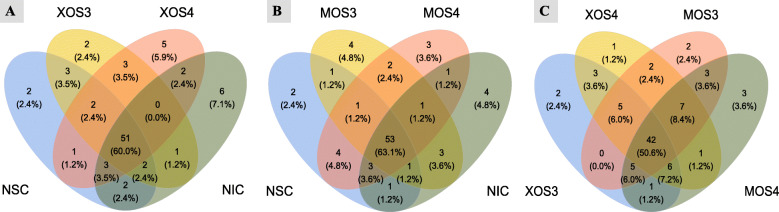
Venn diagram (**A–C**) illustrating the observed overlap of OTUs shared by each treatment group along with the unique OTUs present in each treatment group cecal contents on d 28 post-hatch in response to in ovo injection. NSC: normal saline, XOS3: xylotriose, XOS4: xylotetraose, MOS3: mannotriose, MOS4: mannotetraose, NIC: no injection control

The Venn diagram of the OTUs clustered against the Greengenes reference sequence was generated for XOS3 and XOS4, and MOS3 and MOS4 groups with NSC and NIC groups as well as within those groups (Fig. 5). The Venn diagram showed that 60% OTUs were common among XOS3, XOS4, NSC, and NIC. Similarly, 63.1% OTUs were common among MOS3, MOS4, NSC, and NIC, whereas XOS3, XOS4, MOS3, and MOS4 shared only 50.6% OTUs. The MOS4 group with significantly different OTUs compared with NSC had a trend with NIC in the observed richness of OTUs (Fig. 4). The MOS4 also had 3 unique OTUs and 5 non-overlapping OTUs with both controls. The MOS3 had 3 unique OTUs compared with other oligosaccharides. The XOS4 group had 5, the highest number of unique OTUs compared with XOS3 and both controls, but 4 out of 5 unique OTUs overlapped in the Venn diagram with MOS3 and MOS4 (Fig. 5). Overall, the unique OTUs observed only in one of the oligosaccharides belonged to the following bacterial families (f): f_Clostridiaceae and f_Peptostreptococcaceae in XOS3; f_Clostridiaceae, f_Lactobacillaceae, f_Bacillaceae and f_Corynebacteriaceae in XOS4; f_Streptomycetaceae and f_Brucellaceae in MOS3; f_Lactobacillaceae and f_Erysipelotrichaceae in MOS4.

**Fig. 6:**
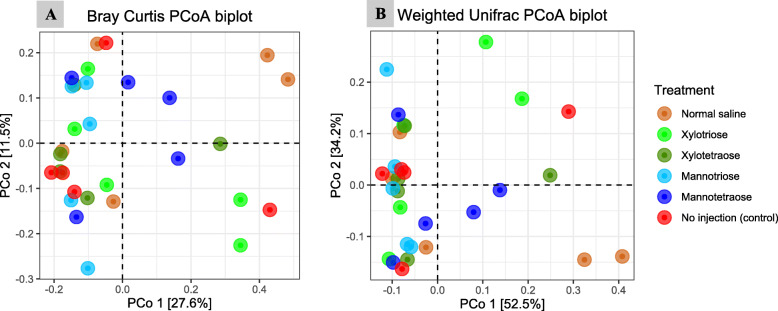
Principal coordinate analysis (PCoA) plot: **A)** Bray Curtis distance, and **B)** Weighted UniFrac distance shows microbiota community beta diversity between different treatments at 20,000 reads depth per sample of cecal contents of broilers from d 28 post-hatch that were administered different treatments through amnion. The x- and y-axes are indicated by the first and second coordinates, respectively, and the values in parentheses show the percentages of the community variation explained. The pairwise comparison did not show any significant differences (*P* > 0.05). The color of the bubble displayed in the legend on the right indicates the respective treatments on the plots

**Fig. 7:**
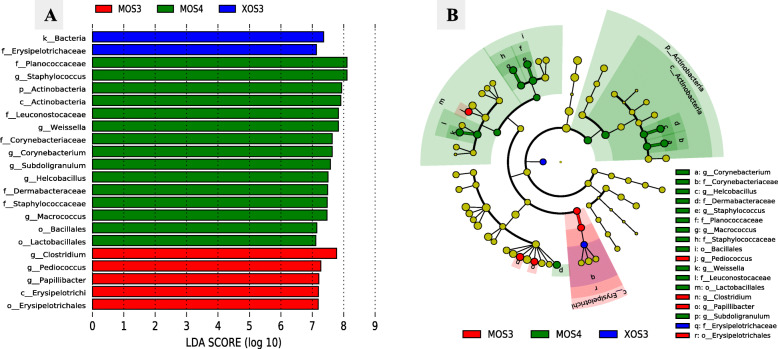
**A)** Histogram shows linear discriminant analysis (LDA) scores of taxa differentially abundant and **B)** Cladogram shows differentially abundant taxa (highlighted by small circles and by shading) at various taxonomic levels between different in ovo treatments in broilers at d 28 post-hatch. Statistical analyses were performed using linear discriminant analysis effect size (LEfSe). MOS3: mannotriose, MOS4: mannotetraose; XOS3: xylotriose

**Fig. 8:**
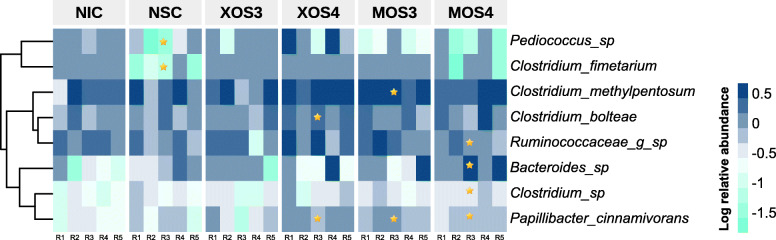
A heatmap showing the log_10_ relative abundance of significantly different cecal microbes in different treatments compared with no injection control in broilers at d 28 post-hatch. The value of abundance is denoted by the color of the heatmap, and the values are represented in the legend, where the light green color denotes the lower abundance while the blue color denotes higher abundance. The yellow asterisks in the cell indicate a statistically significant treatment compared to the no injection control for the bacterial species in the row (*P ≤ 0.05). The dendrogram is generated by the Euclidean distance method and represents the clustering of bacterial species. The column labels R1–R5 denote replicate samples in each treatment. NSC: normal saline, XOS3: xylotriose, XOS4: xylotetraose, MOS3: mannotriose, MOS4: mannotetraose, NIC: no injection control

**Fig. 9:**
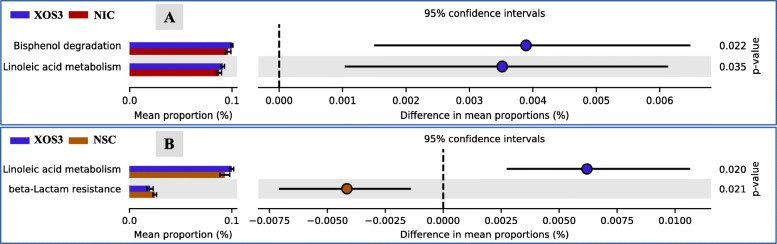
Extended error bar plot showing the mean proportion (%) of predicted functions of cecal microbial communities between **A)** xylotriose and no injection control, and **B)** xylotriose and normal saline at d 28 post-hatch. The *P*-value on the right was derived from a White’s non-parametric t-test in the statistical analysis of taxonomic and functional profiles (STAMP) software. NSC: normal saline control, XOS3: xylotriose, NIC: no injection control

The Bray Curtis and the UniFrac measure of beta diversity for the differential communities were not different (*P* > 0.05) between treatments (Fig. 6). However, further analysis of microbiota revealed that MOS4 enhanced the frequency of differentially abundant (*P* < 0.05) cecal microbial taxa, including order Lactobacillales and family Leuconostocaceae compared with other treatments (Fig. 7). Species-level log relative abundance revealed that *Clostridium* sp. was higher in XOS4, MOS3, and MOS4 groups compared with the NIC group (Fig. 8). The abundance of *Papilibacter cinnamivorans* was higher in XOS4, MOS3, and MOS4 than NIC group (*P* < 0.05). The analysis of the predicted functions of cecal microbial communities revealed that XOS3 had a significantly higher proportion of the mean predicted functions for the linoleic acid metabolism than both controls (*P* < 0.05; Fig. 9). The spearman’s correlation (ρ) among growth performance variables and SCFA in different treatment groups revealed that there was a positive correlation between acetate and ADFI, and propionate and ADG in XOS3 treatment (*P* < 0.05; Table S1; Fig. S1). In the NSC group, butyrate was positively correlated with ADFI and ADG (*P* < 0.05). Compared with other treatment groups, a positive correlation between acetate and FCR was observed in the MOS4 group; however, MOS3 negatively correlated with acetate and FCR (*P* < 0.05).

## Discussion

The beneficial effects of prebiotics feeding on the microbiota diversity and their fermentation metabolites have been well documented in previous studies [2, 3]. However, whether the effects of prebiotics feeding on immunity is via the direct interaction with immune cells or through the produced metabolites and enhanced microbiota becomes confounded during the late phase feeding in broilers when the gut microbiota gets well established [18, 29]. The hatchability was not different across treatments, which conveyed that those treatments had no negative impact on embryos’ livability. The treatments did not influence the growth performance and organ weights. Similar to our finding, Maiorano et al. [30] did not observe any significant improvement in the body weight and FCR of 42 d broilers in response to in ovo injection of trans-galactooligosaccharides. The growth of broilers is more influenced by the density of nutrients in feed in a healthy flock. All the treatment groups were fed the same commercial diet throughout the growth period, and the flocks remained unchallenged. Therefore, improvement in other gut health parameters would not have caused any major influence on the bird’s overall growth performance.

We found that XOS4 could improve the morphological development of intestinal mucosa on hatch day. Ding et al. [5] also recorded the increase in VH and VH:CD ratio in the jejunum of layers with increasing dose of XOS. De Maesschalck et al. [4] supplemented XOS at 0.2 to 0.5% with 2–7 degrees of polymerization, and it increased the ileal villus length in the broilers. However, the improvement in the ileal mucosal histomorphology was not evident after a week and during the later period and thus indicates that the stimulant action on ileal mucosa would have been removed with the growth and the passage of feed. The increased production of acetate and butyrate in the XOS3 group is in line with the findings of Ding et al. [5], who reported that the addition of XOS increased butyrate and, to some extent, raised acetate in the ceca of layers. In broilers, Pourabedin et al. [31] also observed the increase in propionate besides butyrate in response to XOS addition in feed. However, in agreement with our findings, Yuan et al. [32] also reported that the supplementation of XOS increased both acetate and butyrate in the ceca of broilers. In contrast with the effect of XOS3 on the ileal mucosa, its influence on the production of SCFA during the later growth phase of broilers infer to its ‘stimbiotic’ role on gut microbiota [33]. The variability in the fermentation characteristics of XOS could be reasonable because of the inconsistency in the composition of oligomer residues containing a different degree of polymerization. This could also be true for the current study as we did not detect the same level of SCFA production in the XOS4 group as observed in the XOS3 group. The increased ratio of VH:CD in the MOS3 treatment compared with NIC corresponds well with the study of Baurhoo et al. [34] in broilers, where MOS had increased the VH in the jejunum. Pourabedin et al. [35] found that MOS increased the VH in both ileum and jejunum of male broilers. Moreover, the addition of MOS in the broiler feed effectively increased the intestinal VH in the studies of Chee et al. [36] and Micciche et al. [37].

Besides the absorptive cells, the broilers intestine also contains gut-associated lymphoid tissue (GALT), consisting of immune cells such as T and B cells. It has been realized that such immune cells can be modified by diet and by the intestinal microbiota [38, 39], which could be a consequence of activation of dendritic cells in Peyer’s patches that would then stimulate the circulating T-lymphocytes [40]. The XOS3 has also displayed its potential to stimulate early cell-mediated immunity and inflammatory response. The XOS3 treatment groups had a trend for a reduced level of *IL-10* on the hatch day compared with both controls. The *IL-10* is a potent anti-inflammatory cytokine, and its impaired level could exaggerate inflammatory response; however, such signaling can also promote the clearance of pathogens during acute infection [41]. The supplemental XOS in the study of Pourabedin et al. [42] has also been reported to stimulate the immune system and reduce *Salmonella* colonization. Akhtar et al. [43] verified that arabinoxylooligosaccharides possess the immunomodulatory capacity to stimulate the humoral immune response in chickens. However, we observed variability in the immune activation by two different XOS oligomers in the present study. Zhou et al. [44] compared immunological activities of arabinoxylan extracted from wheat bran, and the results suggest that the immunological properties of arabinoxylan are affected by the chemical composition, molecular weight, and degree of arabinose substitution and branching. The increase in the gene expression of T cells by the XOS3 is interesting to note as Toll-like receptors and dendritic cells have been known to recognize the commensal bacteria and lead to the differentiation of regulatory T cells [45]. Thus, further evaluation of the immunological properties of XOS of different polymer lengths is warranted in early and subsequent feeding phases. The effect of MOS4 on the *IL-10* should also be considered as an important immune response as the MOS supplementation in the broilers has been stated to provide an adjuvant-like effect by mimicking microbial antigen [37]. Similar to the current study’s findings, Madej and Bednarczyk [46] found that in ovo injection of trans-galactooligosaccharides prebiotics increased T cells colonization in cecal tonsils at d 7, but the effect was lesser on d 21. The early effect of prebiotics on immune cells is expected to be their direct effect on intestinal cells rather than being mediated through gut microbiota as the embryo’s intestine is considered sterile, and it takes some time for the establishment of stable gut microbiota [11, 12].

The analysis of cecal microbiota showed that the Firmicutes dominated the bacterial phyla and ranged around 75% while the Bacteriodetes was second most dominant with a range of around 10–20% that was also reported previously [4]. The LEfSe elucidated that the MOS3 group had a higher proportion of genus *Pediococcus* belonging to the family Lactobacillaceae capable of producing lactic acid and genus *Papillibacter* belonging to the family Ruminococcaceae that produces enzymes to degrade and utilize lignin in dietary fiber [1, 47]. Likewise, the MOS4 group had a higher abundance of family Leuconostocaceae, a group of lactic acid-producing bacteria. Thus, the enhanced proportion of these beneficial bacteria by these oligosaccharides is expected to provide prebiotic benefit and improve the intestinal health of broilers.

The gut microbiota plays a major role in maintaining normal physiology in the intestine beyond substrate fermentation. It appears that the XOS3 group had some notable microbial pathways predicted by the PICRUSt that were altered compared with the controls. The linoleic acid metabolic pathways have been documented as an important link in the development of inflammation [48]. The enrichment of this metabolic pathway along with the amplification of T cell gene expression and the decline in the *IL-10* gene expression in the XOS3 group suggest their association in inflammation; however, it is not clear whether it would be a desirable outcome. Additionally, the increased level of microbial bisphenol degradation pathway in the XOS3 group is expected to reduce bisphenol, which has an important role in reducing toxicity and estrogenic activity [49]. Moreover, Spearman’s correlation in the XOS3 group suggests that higher propionate production supports the growth of the birds, and this accretion would be in the form of other tissues rather than as fat deposition [50].

The in ovo administration of the oligosaccharides in this study did not differ the growth performance, and the outcome as such is not conclusive to support the application of these oligomers exclusively as a non-antibiotic growth promoter. To our knowledge, this is the first study that evaluated the effects of in ovo feeding of XOS and MOS differing in the degree of polymerization on the intestinal morphological parameters, immunity, and the gut microbiota of broiler chicken. We observed a more prominent effect of XOS3 treatment on immune cells and SCFA production than other oligosaccharides, whereas the MOS3 and MOS4 had a major influence on microbiota diversity in the ceca of broilers. Thus, it is plausible to accept that a variation exists in the mechanism of these prebiotics in impacting the health status of broilers. Moreover, further investigations on the combined use of XOS3 and MOS4 are necessary to define the complementary effects of these prebiotics. This study indicates that the effect of XOS3 on immunity is transient, but its effect on cecal fermentation is prolonged.

## Conclusion

This study provided valuable insights into the functioning of prebiotics (oligosaccharides differing in the degree of polymerization) when introduced in the GIT of broilers through in ovo feeding. It is intriguing to note that the enhancement of immunity during hatch must be the direct effect of oligosaccharides as the cecal SCFA production would only become dominant after the stabilization of the cecal microbiota. The in ovo injection of XOS and MOS did not affect the growth performance of the broilers, but the relative weight of the gizzard was lowest in XOS3 and highest in MOS4 groups. The VH:CD ratio was increased in the XOS4 group compared with both controls (NSC and NIC). On d 28, XOS3 increased total SCFA production, including the levels of acetate and butyrate in ceca. Moreover, XOS3 increased the gene expression of *CD3* (a T cell marker) on hatch day, whereas MOS4 decreased the gene expression of *IL-10* (a marker of anti-inflammatory cytokine). In addition, MOS4 increased the frequency of observed OTUs compared with NSC and enhanced the abundance of order Lactobacillales and family Leuconostocaceae in ceca of broilers on d 28 compared with all treatments. The number of eggs included in this study in each treatment unit was low, and some observed differences in the variables did not reach significance. Thus, more in ovo investigation would be necessary to understand the transient immune response in broilers to the oligosaccharides administration. Further research on the mechanism and potential of these prebiotic products is warranted before their extensive use alone or in combination with other additives as an alternative to AGP.

## Supplementary Information

**Additional file 1 **Table S1 Spearman’s correlation between growth performance parameters and major short-chain fatty acids in each treatment in broilers at d 28 post-hatch. Fig. S1 Biplot (individual samples scatter removed) from principal component (PC) analysis of growth performance parameters and the major cecal short-chain fatty acids showing correlations among these variables in different treatment groups in two PC (PC 1 and PC 2). The PC percentage on the x and y-axis indicates the proportions of the variability of the data explained by those components. The angle between the loading arrows and their directions indicates negative, positive, or no association, while the length indicates the value of correlation (e.g., α = 0° and *r* = 1; α = 90° and *r* = 0; and α = 180° and *r* = − 1). See Table S1 for the significant spearman’s correlation coefficient. ADFI: average daily feed intake, ADG: average daily gain, FCR: feed conversion ratio, FBW: final body weight. NSC: normal saline, XOS3: xylotriose, XOS4: xylotetraose, MOS3: mannotriose, MOS4: mannotetraose, NIC: no injection control.

## Data Availability

None.

## Abbreviations

ADFI: Average daily feed intake
ADG: Average daily gain
AGP: Antibiotic growth promoters
CD3: Cluster of differentiation 3
CD56: Neural cell adhesion molecule (NCAM)
chB6: Chicken B-cell marker (Bu-1)
FCR: Feed conversion ratio
IL: Interleukin
MOS: Mannan-oligosaccharides
OTU: Operational taxonomic unit
SCFA: Short-chain fatty acids
TLR: Toll-like receptor
XOS: Xylo-oligosaccharides
