# Supplementary material for: Effects of in ovo delivered xylo- and mannan- oligosaccharides on growth performance, intestinal immunity, cecal short-chain fatty acids, and cecal microbiota of broilers
Source: J Anim Sci Biotechnol. 2022 Feb 8;13:13. doi: 10.1186/s40104-021-00666-z (PMC8822640; doi:10.1186/s40104-021-00666-z)
Supplement: Supplementary file 1 — Additional file 1 Table S1 Spearman’s correlation between growth performance parameters and major short-chain fatty acids in each treatment in broilers at d 28 post-hatch. Fig. S1 Biplot (individual samples scatter removed) from principal component (PC) analysis of growth performance parameters and the major cecal short-chain fatty acids showing correlations among these variables in different treatment groups in two PC (PC 1 and PC 2). The PC percentage on the x and y-axis indicates the proportions of the variability of the data explained by those components. The angle between the loading arrows and their directions indicates negative, positive, or no association, while the length indicates the value of correlation (e.g., α = 0° and r = 1; α = 90° and r = 0; and α = 180° and r = − 1). See Table S1 for the significant spearman’s correlation coefficient. ADFI: average daily feed intake, ADG: average daily gain, FCR: feed conversion ratio, FBW: final body weight. NSC: normal saline, XOS3: xylotriose, XOS4: xylotetraose, MOS3: mannotriose, MOS4: mannotetraose, NIC: no injection control. [file 40104_2021_666_MOESM1_ESM.zip › Suppl Table 1.docx]

**Supplementary Information**

**Table S1** Spearman correlation between growth performance parameters and major short chain fatty acids in each treatment in broilers at d 28 post-hatch.

|  |  | Treatments | | | | | | | | | | | | | | | |
| --- | --- | --- | --- | --- | --- | --- | --- | --- | --- | --- | --- | --- | --- | --- | --- | --- | --- |
|  |  | Normal Saline | | | No injection | | | Xylotriose | | | Xylotetraose | | | Mannotriose | | Mannotetraose | |
| Variable | By variable | ρ | *P*>\|ρ\| | ρ | | *P* >\|ρ\| | ρ | | *P* >\|ρ\| | ρ | | *P* >\|ρ\| | ρ | | *P* >\|ρ\| | ρ | *P* >\|ρ\| |
| ADG | ADFI | 0.90 | 0.037 |  | |  |  | |  |  | |  | **0.90** | | **0.037** |  |  |
| FCR | ADFI | -0.90 | 0.037 |  | |  |  | |  |  | |  |  | |  |  |  |
| FCR | ADG | -1.00 | <0.001 |  | |  |  | |  |  | |  |  | |  |  |  |
| FBW | ADFI | 0.90 | 0.037 |  | |  |  | |  |  | |  | **0.90** | | **0.037** |  |  |
| FBW | ADG | 1.00 | <0.001 | 1.0 | | <0.001 | 1.0 | | <0.001 | 0.97 | | 0.005 | 1.00 | | <0.001 | 1.00 | <0.001 |
| FBW | FCR | -1.00 | <0.001 |  | |  |  | |  |  | |  |  | |  |  |  |
| Acetate | ADFI |  |  |  | |  | **0.9** | | **0.037** |  | |  |  | |  |  |  |
| Acetate | FCR |  |  |  | |  |  | |  |  | |  | **-0.90** | | **0.037** | **0.90** | **0.037** |
| Propionate | ADFI | -0.90 | 0.037 |  | |  |  | |  |  | |  |  | |  |  |  |
| Propionate | ADG |  |  |  | |  | **0.9** | | **0.037** |  | |  |  | |  |  |  |
| Propionate | FBW |  |  |  | |  | **0.9** | | **0.037** |  | |  |  | |  |  |  |
| Butyrate | ADFI | 0.90 | 0.037 |  | |  |  | |  |  | |  |  | |  |  |  |
| Butyrate | ADG | 1.00 | <0.001 |  | |  |  | |  |  | |  |  | |  |  |  |
| Butyrate | FCR | -1.00 | <0.001 |  | |  |  | |  |  | |  |  | |  |  |  |
| Butyrate | FBW | 1.00 | <0.001 |  | |  |  | |  |  | |  |  | |  |  |  |
| Butyrate | Acetate |  |  |  | |  |  | |  | **0.90** | | **0.037** |  | |  |  |  |
| Butyrate | Propionate |  |  |  | |  |  | |  |  | |  |  | |  | **0.90** | **0.037** |
| Total SCFA | FCR |  |  |  | |  |  | |  |  | |  | **-0.90** | | **0.037** |  |  |
| Total SCFA | Acetate | 1.00 | <0.001 |  | |  |  | |  | 1.00 | | <0.001 | 1.00 | | <0.001 | 0.90 | 0.037 |
| Total SCFA | Propionate |  |  |  | |  |  | |  |  | |  |  | |  | **0.90** | **0.037** |
| Total SCFA | Butyrate |  |  |  | |  |  | |  | **0.90** | | **0.037** |  | |  |  |  |

ρ (rho): Spearman correlation coefficient, ADFI: average daily feed intake, ADG: average daily gain, FCR: feed conversion ratio, FBW: final body weight, SCFA: short chain fatty acids. Only significant correlations are shown for each treatment. Each treatment had 5 replicates, and hence only highly significant correlation has a meaningful interpretation.
